# Quantification of branching within high molecular weight polymers with polyester backbones formed by transfer-dominated branching radical telomerisation (TBRT)†

**DOI:** 10.1039/d1ra03886a

**Published:** 2021-07-12

**Authors:** Savannah R. Cassin, Sean Flynn, Pierre Chambon, Steve P. Rannard

**Affiliations:** Department of Chemistry, University of Liverpool Crown Street L69 7ZD UK srannard@liv.ac.uk; Materials Innovation Factory, University of Liverpool Crown Street L69 7ZD UK

## Abstract

New branched polymerisations offer previously inaccessible macromolecules and architectural understanding is important as it provides insight into the branching mechanism and enables the determination of structure–property relationships. Here we present a detailed inverse gated ^13^C NMR characterisation of materials derived from the very recently reported Transfer-dominated Branching Radical Telomerisation (TBRT) approach to quantify branching and provide an insight into cyclisation.

## Abstract 2

The characterisation and quantification of branching is key to understanding new complex macromolecules. Here we establish approaches to evaluate the unique and novel architectures formed by Transfer-dominated Branching Radical Telomerisation (TBRT).

## Graphical abstract

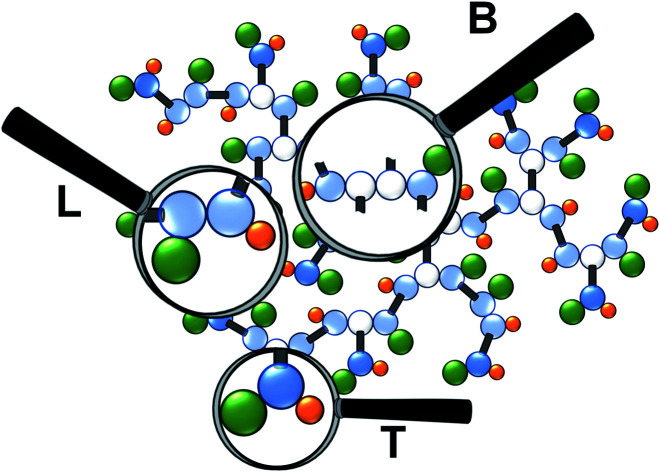

## Introduction

The synthesis of branched polymers has intrigued polymer chemists from the earliest days of polymer science including establishing paradigms for their synthesis and characterisation,^1,2^ controlling their structure,^3–5^ avoiding gelation,^6,7^ theoretical considerations^8^ and methods for their scale-up^9–11^ and application.^12,13^ Several excellent reviews^14–16^ have been published that highlight the versatility of this polymer topology, and the benefits and complexities of branched architectures that may range from perfectly branched and monodisperse dendrimers through to macromolecules that are essentially linear in their nature and have low branching densities.

Branched polymer synthesis strategies can be simply divided into two categories of resulting products: (1) materials that have backbones that resemble step-growth polymers (heteroatoms within the backbone); and (2) materials with predominantly C–C backbone chemistries.^17–19^ Although a slight over-simplification, branched materials with backbones resembling step-growth chemistries are most often produced using AB_*n*_ monomers and employ conventional step-growth chemistries such as esterification and amide formation.^20^ Notable exceptions from this simplified description include ring-opening branched polymerisations forming materials such as polyglycerol^21,22^ and polyethylene imine.^23^

**Fig. 1:**
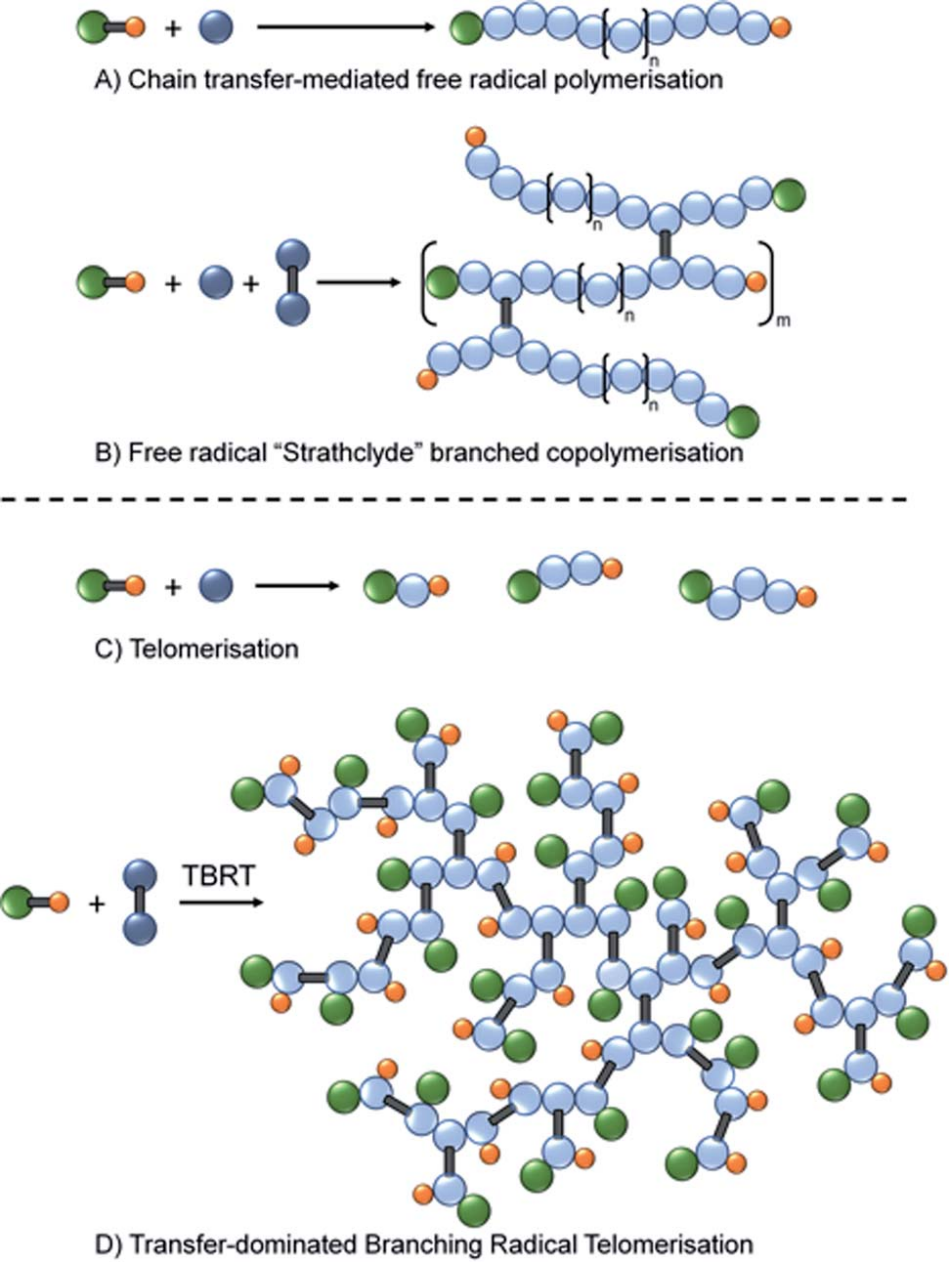
Schematic representation of (A) synthesis of linear polymers by conventional free radical polymerisation in the presence of chain transfer agents; (B) “Strathclyde” strategy for generating branched vinyl copolymers using low concentrations of multi-vinyl monomers; (C) telomerisation using a high chain transfer agent (telogen) : monomer (taxogen) molar ratio; (D) transfer-dominated branching radical telomerisation of a multi-vinyl taxogen.

Within the categorisation of branched polymers with predominantly C–C backbones, the use of multi-vinyl monomers in a copolymerisation with monovinyl monomers has become a popular strategy, Fig. 1. Initially reported by Sherrington and coworkers,^24^ and termed the “Strathclyde route”, the first reports of high molecular weight polymers formed in the absence of gelation utilised conventional free radical approaches with thiol chain transfer agents used to control the length of the primary chains within the architecture, Fig. 1A and B.^25^

Importantly, the frequency of multi-vinyl monomer incorporation was also controlled to ensure less than one branch per primary chain on average, Fig. 1B.^26^ Controlled polymerisations have also been shown to benefit from restricting multi-vinyl monomers to less than one per primary chain^27^ and polymers have been reported that utilise techniques such as ambient temperature anionic polymerisation,^28^ group transfer polymerisation,^29^ oxyanionic polymerisation,^30^ atom transfer radical polymerisation (ATRP)^31,32^ and reversible addition fragmentation chain transfer (RAFT)^33^ chemistries. Other strategies that generate branched polymers with predominantly C–C backbones include self-condensing vinyl polymerisation^34,35^ and anionic polymerisation^36^ to form hyperbranched structures, and ideally branched architectures.^37^

The homopolymerisation of multi-vinyl monomers is intrinsically complicated by the potential for network formation and the synthesis of insoluble gels. In recent years, controlled polymerisations such as catalytic chain transfer polymerisation,^38,39^ RAFT,^40^ and deactivation enhanced ATRP^41^ have been employed in the formation of branched polymers containing residual vinyl groups after the polymerisation of divinyl monomers such as ethylene glycol dimethacrylate (EGDMA) or divinyl benzene. The complete conversion of monomer vinyl bonds was not achieved in these reports and the reactions were terminated prior to high conversion to prevent gelation and conducted at <40 wt% solids content (RAFT and ATRP).

Recently we reported a new synthetic approach to highly branched polymer synthesis that utilises conventional free radical telomerisation approaches, namely Transfer-dominated Branching Radical Telomerisation (TBRT).^42^ The earliest clear reports of telomers and telomerisation are to be found in the patent literature between 1945–1946,^43–47^ with a comprehensive review published in 1952.^48^ In summary, telomers are very short polymer chains with a number average degree of polymerisation (DP_*n*_) usually <5 monomer units, Fig. 1C. Importantly, and under free radical conditions, telomerisation often utilises a Y–X molecule (a telogen) to add across an unsaturated double bond Z (the taxogen) to create an adduct Y–(Z)_*n*_–X (the telomer), where *n* < 5.^49^ This is similar to the use of a chain transfer agents (CTA) in a CTA-mediated free radical polymerisation, Fig. 1A; however, telomerisation requires higher concentrations of CTA (telogen) in order to deliver maximum control of propagation, Fig. 1C.

**Scheme 1:**
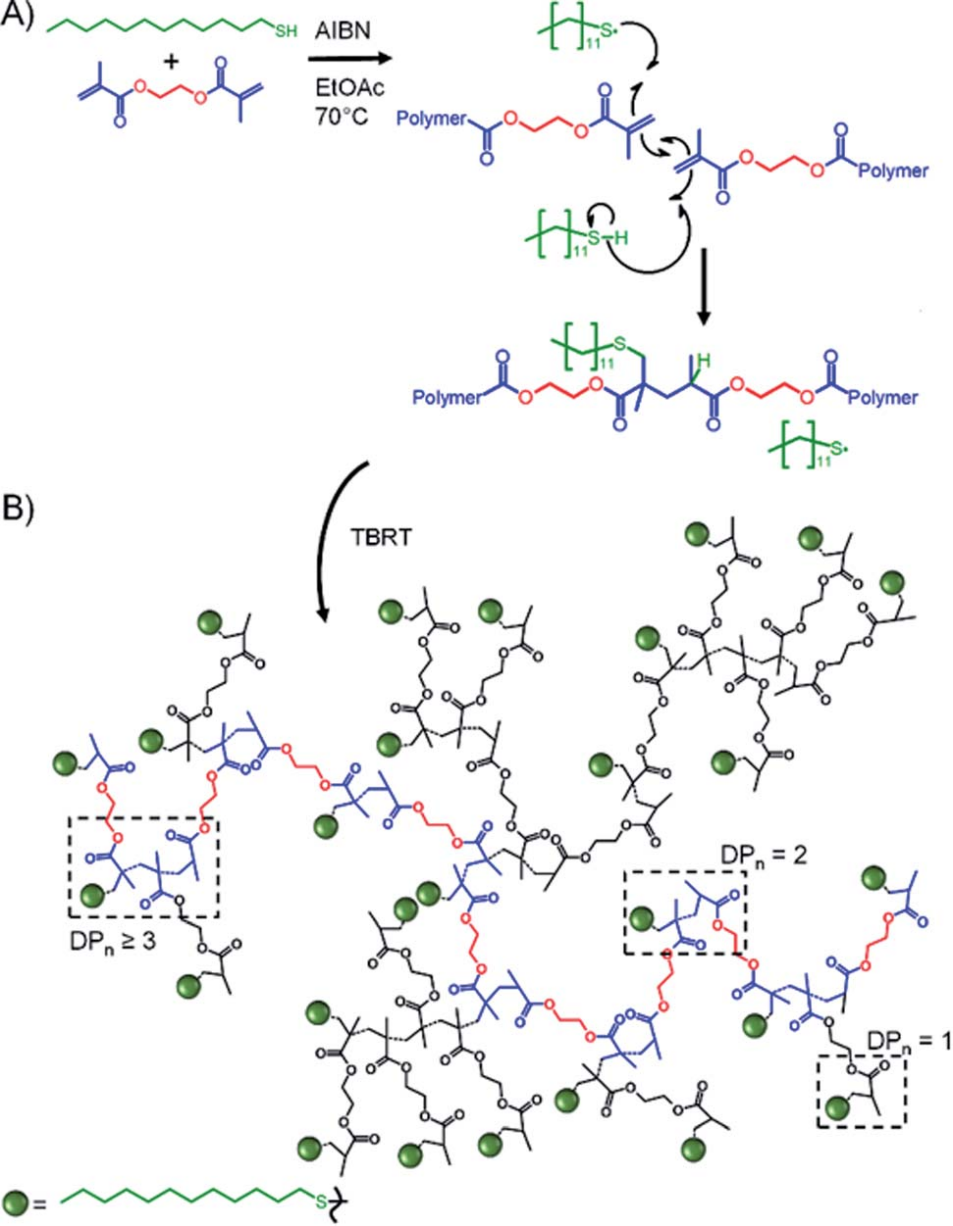
Simplified overview of Transfer-dominated Branching Radical Telomerisation (TBRT). (A) Reaction of EGDMA taxogen with 1-dodecane thiol (DDT) telogen under telomerisation conditions showing DDT radical addition and limited propagation (number average < 2) with rapid transfer to telogen; (B) branched thioether containing polyester TBRT product.

TBRT employs the reaction of telogens with multi-vinyl taxogens to ensure a number average propagation of <2 vinyl groups within the telomerisation reaction, Fig. 1D, complete vinyl conversion and no observed gelation, Scheme 1. The telomers that result are covalently ‘linked’ by the chemistry of the multi-vinyl monomer and the resulting high molecular weight polymer is therefore not comprised of extended C–C backbones, Scheme 1A; for example, the homo-telomerisation of EGDMA leads to a thioether containing branched polyester, Scheme 1B. From one perspective, it is analogous to a controlled C–C bond formation within the multi-functional acid monomer A residues of an A_*n*_ + B_2_ branched copolymerisation,^50^Scheme 1A. TBRT, therefore, allows the formation of branched polymers comprising step-growth chemistries by employing conventional free radical reactions of multi-vinyl monomers.^42^

The characterisation and theoretical understanding of branched polymers is well known to be complex and various techniques have been reported for modelling and measuring these architectures. This includes studying the development of branched structures,^51^ branching density^52^ or calculating degree of branching^53,54^ or average number of branches.^55^ Techniques such as size exclusion chromatography,^56–59^ light scattering^60^ techniques, thermal field-flow fractionation,^61,62^ hydrolysis/analysis^63^ of branched structures and measurement using advanced nuclear magnetic resonance spectroscopy (NMR) techniques^64,65^ of either the direct polymerisation product or after post-polymerisation reaction of unreacted functional groups^66,67^ have been reported for a wide range of material chemistries. The factors that confound accurate branching evaluation distribution include the overlap of critical NMR resonances and the presence of a distribution of architectures within the polymer molecular weight distribution.

Additionally, the potential for macrocycle formation within the complex branched structures generates further complexity.^68^ Modelling of cycle formation has been achieved for AB_2_ polymerisations^69^ and modified “Strathclyde” polymerisations;^51^ however, identification of large cyclic substructures in hyperbranched molecules is particularly difficult and is not readily achieved using techniques commonly applied to single macrocycle identification within predominantly linear polymer samples. Complex NMR studies, possibly coupled to time-of-flight matrix assisted laser desorption ionisation mass spectrometry, is often utilised to quantify a lack of structural or functional components (for example, the focal point chemistry of an AB_2_ hyperbranched polymer) and hence indicate intramolecular reaction leading to cyclisation.^70^

As mentioned above, the determination of branching within complex polymer architectures is important as it allows an understanding of the impact of reaction variables on architecture and the impact of architecture on sample properties. Herein, we report a detailed and readily accessible inverse gated ^13^C NMR approach that allows the quantification of specific carbon environments and a direct estimation of the percentage of vinyl bonds contributing to linear, branched and terminal units within the branched polymers. This has been monitored for varying reaction conditions and, by utilising solvent fractionation to separate different fractions of the polymer sample, the degree of branching has been correlated to molecular weight and weight fraction.

## Results and discussion

**Fig. 2:**
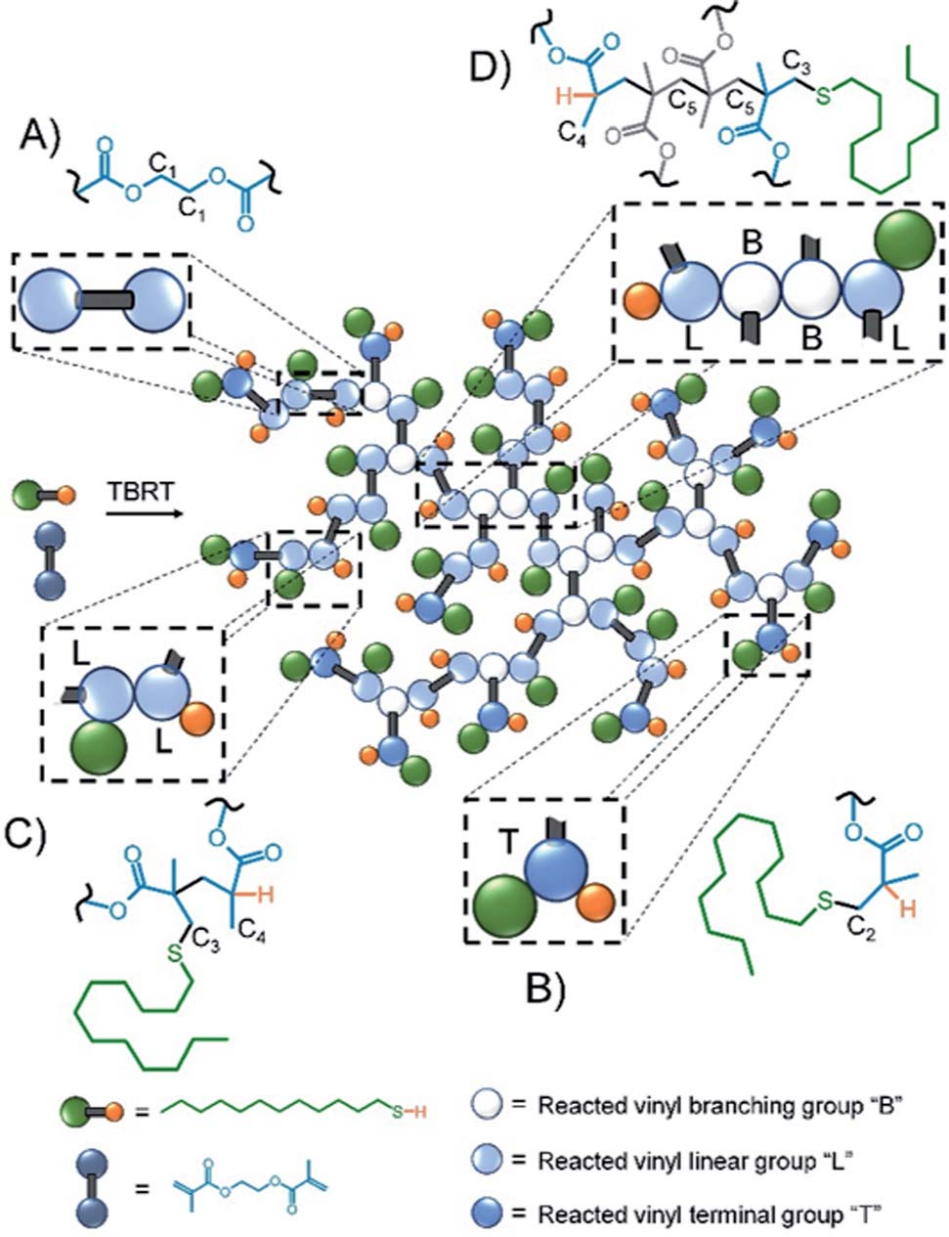
Schematic representation of TBRT polymer and structural units comprising (A) EGDMA residue; (B) terminal (DP_*n*_ = 1) groups; (C) linear (DP_*n*_ = 1) groups and (D) branching (DP_*n*_ > 2) units.

Inverse gated ^13^C NMR spectroscopy has been employed previously for structural analysis of branched polymers formed by self-condensing vinyl polymerisation under ATRP microemulsion polymerisation conditions.^71,72^ The structures of polymers resulting from TBRT are fundamentally different to those formed by the homopolymerisation of inimers and a relatively simple approach was adopted to calculate the molar ratio of the different contributions of the reacted vinyl groups to branching, linear and terminal structures within the complex architecture. Importantly, in our study we have defined a DP_1_ structure, shown chemically in Scheme 1B and schematically in Fig. 2, as a terminal group, T. Reacted vinyl groups that contribute to linear segments, L, of the polyester are defined as the two units of a DP_2_ telomer and the two end groups of any telomer with DP_*n*_ > 2 units; reacted vinyl groups within a telomer of DP ≥ 3 units are defined as branch points, B, Fig. 2. The inverse gated quantitative ^13^C NMR spectra of TBRT polymers derived from EGDMA (taxogen) and dodecanethiol (DDT; telogen) present several resonances that are unaffected by overlap from other carbon environments; these also allow quantification of key structural units, Fig. 2. It is well known that the relaxation of substituted carbons may vary due to different levels of substitution,^73^ therefore we have selected to use only methylene carbon resonances for our studies. Within the polyester backbone, the methylene carbons derived from ethylene glycol units (carbon C_1_ at *δ* = 62.3 ppm, Fig. 2A) are readily distinguishable and equate to the total number of vinyl group residues throughout the TBRT polymer. The DP_1_ terminal vinyl residues have several distinct resonances that are unique to the terminal units and integrate equally; the methylene carbon C_2_ at *δ* = 34.5 ppm, Fig. 2B, establishes the number of terminal units relative to the overall number of vinyl residues. Simple subtraction leads to the determination of the number of vinyl residues that constitute internal telomer structures (*i.e.* linear and branched units). The methylene carbon C_3_ at *δ* = 35.5 ppm, Fig. 2C and D, resulting from telogen addition at the α chain-end of each telomer (*i.e.* DP_*n*_ ≥ 2 units) is clearly equal in number to the ω-chain ends (C_4_, Fig. 2C and D). Here we define the α and ω chain ends as linear units, L, irrespective of the chain length of the telomer as they independently do not lead to a branch point. As the integral of C_3_ is very distinct within the spectrum, simply doubling this integral will therefore account for all vinyl residues contributing to linear groups; subtraction will lead to the number of reacted vinyl residues contributing to branching (*e.g.* methylene C_5_, Fig. 2D). This approach to determine the relative molar ratio of L, T and B vinyl residues has been assessed using different resonances and shown to provide consistent values.

The molecular weight of TBRT polymers increases with increasing taxogen : telogen ratios (*i.e.* increasing EGDMA with respect to DDT); it is important, therefore, to establish the impact of systematically increasing taxogen on branching and how this varies across the molecular weight distribution.

**Fig. 3:**
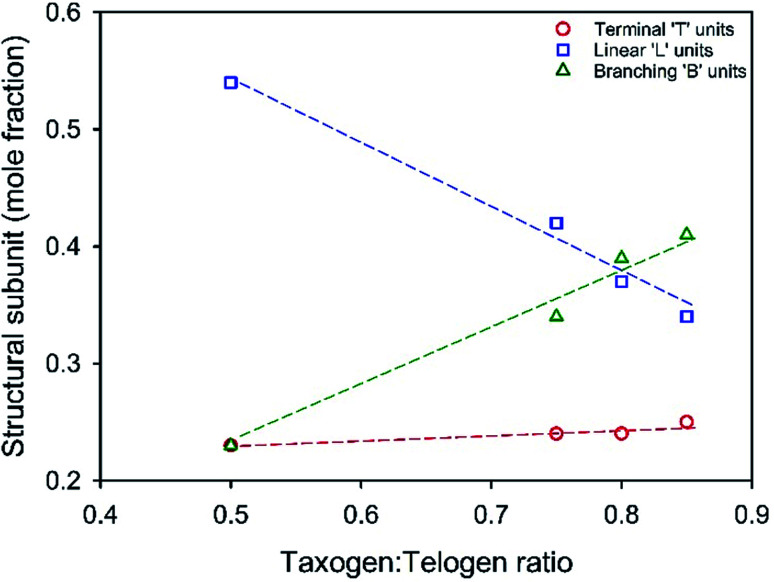
Variation of structural units within TBRT polymers derived from EGDMA (taxogen) and DDT (telogen) with systematic variation of taxogen : telogen ratios in the reaction mixture. Values determined by inverse gated ^13^C NMR analysis.

A series of TBRT reactions utilising taxogen : telogen ratios of 0.50, 0.75, 0.80 and 0.85 were therefore conducted using ethyl acetate as the reaction solvent at 50 wt% solids content. Each reaction reached >99% conversion of vinyl groups as determined by ^1^H NMR of the crude reaction mixture, and taxogen : telogen ratios of 0.85, 1.02, 0.98 and 1.00 were determined for the final purified products (ESI Fig. S1–S3†). Triple-detection size exclusion chromatography (TD-SEC) showed the expected increase in number and weight average molecular weights (*M*_n_, *M*_w_) with *M*_w_ varying from 12 245 to 129 350 to 354 230 and 2 350 000 g mol^−1^ respectively, Table 1 (ESI Fig. S4†). Inverse gated ^13^C NMR analysis of each polymer showed a clear trend in the estimation of reacted vinyl groups leading to T, L and B units, Fig. 3 (ESI Fig. S5–S8†). Interestingly, the mole fraction of T units remained remarkably consistent across the taxogen : telogen ratios; however, a near linear decrease in L units and consequent near linear increase in reacted vinyl groups leading to branching, B units, was observed.

**Table 1:** Detailed analysis of the TBRT of EGDMA with DDT at 70 °C in EtOAc at 50 wt% (initiator: 1.5 mol% AIBN based on vinyl bonds)

NMR (CDCl_3_)						TD-SEC (THF/TEA)^e^				
Taxogen_0_ : telogen_0_^a^	Conv.^b^ (%)	Taxogen_F_ : telogen_F_^c^	B units^d^ (%)	L units^d^ (%)	T units^d^ (%)	*M*_w_ (g mol^−1^)	*M*_n_ (g mol^−1^)	*D*	*A*	dn/dc
0.50	>99	0.85	23	54	23	12 245	4083	3.00	0.288	0.085
0.75	>99	1.02	34	42	24	129 350	4539	28.5	0.294	0.089
0.80	>99	0.98	39	37	24	354 230	5839	60.7	0.334	0.099
0.85	>99	1.00	41	34	25	2 350 000	21 381	110	0.348	0.087
0.90	Gel	—	—	—	—	—	—	—	—	—

a Determined by ^1^H NMR of *t*_0_ sample.

b Determined by ^1^H NMR of crude sample after 24 h.

c Determined by ^1^H NMR of purified and dried material.

d Determined by inverse-gated ^13^C NMR of the purified and dried material and eqn (S4).

e Determined by triple-detection size exclusion chromatography in THF/TEA.

The degree of branching is often calculated using a ratio of L, T and B groups using equations originally reported by Hawker *et al.*^53^ and later modified by Hölter *et al.*^55^ to account for different branching multiplicities within AB_*n*_ polymerisations. As such, and with the understanding that the distribution of telomer lengths within the branched structures is not readily discernible and does not correlate to AB_*n*_ polymerisations, a pragmatic approach is to focus on the mol% of vinyl residues within the polymer that contribute to branching, *i.e.* the mole fraction of EGDMA vinyl groups that have propagated between the α and ω telomer chain ends. This can be simply determined using eqn (1) and (2).1
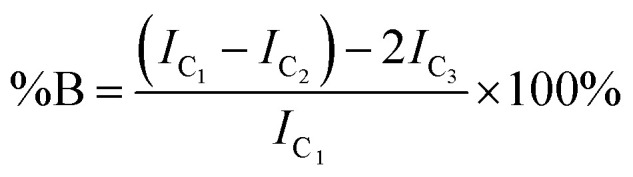
2
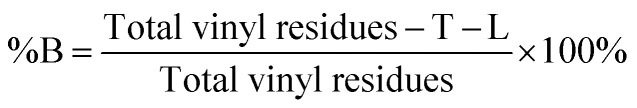


As the taxogen : telogen ratio increases from 0.50–0.85, the B residues increase from 23 mol% to 41 mol% of the total reacted vinyl groups. This is readily rationalised by an increasing kinetic chain length within the free radical telomerisation and a distribution of telomer substructures of increasing chain length.

As mentioned above, branched polymers are known to present an overlapping distribution of molecular weight and architectural complexity. The branching is therefore not expected to be uniform throughout the molecular weight distribution, especially as increasing molecular weight *via* intermolecular reaction is seen throughout the conversion of vinyl groups to polymer;^42^ at high conversion, the concentration of unreacted vinyl groups is low and the pendant functional groups are highly restricted both sterically and in their diffusion/mobility. In previous reports, polymers generated by modified “Strathclyde” approaches utilising ATRP have been separated into distinct fractions using solvent dialysis approaches.^74^

**Fig. 4:**
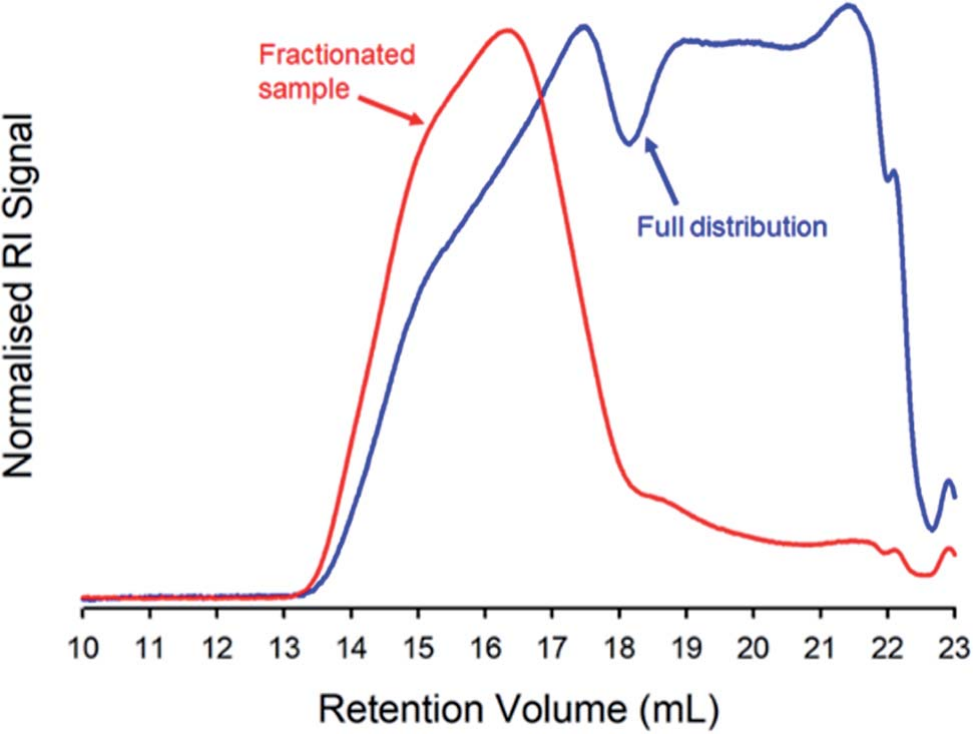
Triple-detection size exclusion chromatography (THF eluent; refractive index (RI) detector signal) showing the molecular weight distribution of a TBRT polymer derived from EGDMA (taxogen) and DDT (telogen) at a taxogen : telogen ratio of 0.85 : 1.00. The overlaid chromatograms show the distributions of the recovered and purified polymer (blue) and the high molecular weight fraction obtained after solvent fractionation (red).

Here, we chose to quantify branching within the highest molecular weight fraction of the TBRT polymer resulting from the taxogen : telogen ratio of 0.85 : 1.00. A solvent fractionation was conducted,^75^ due to the high *M*_w_ observed within this sample. The purified sample was dissolved in THF at a concentration of 100 mg mL^−1^ and an antisolvent (acetone) was added at ambient temperature. The material that precipitated under these conditions was collected and TD-SEC analysis of the high molecular weight fraction exhibited an *M*_w_ = 6 560 000 g mol^−1^ (*M*_n_ = 1 102 000 g mol^−1^) which overlayed almost perfectly with the high molecular weight region of the unfractionated polymer sample (*M*_w_ = 2 350 000 g mol^−1^; *M*_n_ = 21 380 g mol^−1^), Fig. 4 (ESI Table S1 and Fig. S10†).

Inverse gated ^13^C analysis (CDCl_3_; Fig. S11†) of this fraction led to a significant difference in the observed values of the L, T and B groups when compared to the analysis of the full distribution (L = 45 mol%, T = 12 mol%, B = 43 mol%). The potential for restriction of motion leading to suppression of resonances was investigated by conducting the analysis at ambient and elevated temperature (50 °C) using 1,1,2,2-tetrachloroethane-d_2_; however, no variation in resolution was observed (Fig. S13 and S14†). ^1^H NMR analysis (CDCl_3_; Fig. S11†) also revealed a significant variation in the taxogen : telogen ratio within the high molecular weight fraction (1.26 : 1.00) and the unfractionated sample (1.00 : 1.00).

**Fig. 5:**
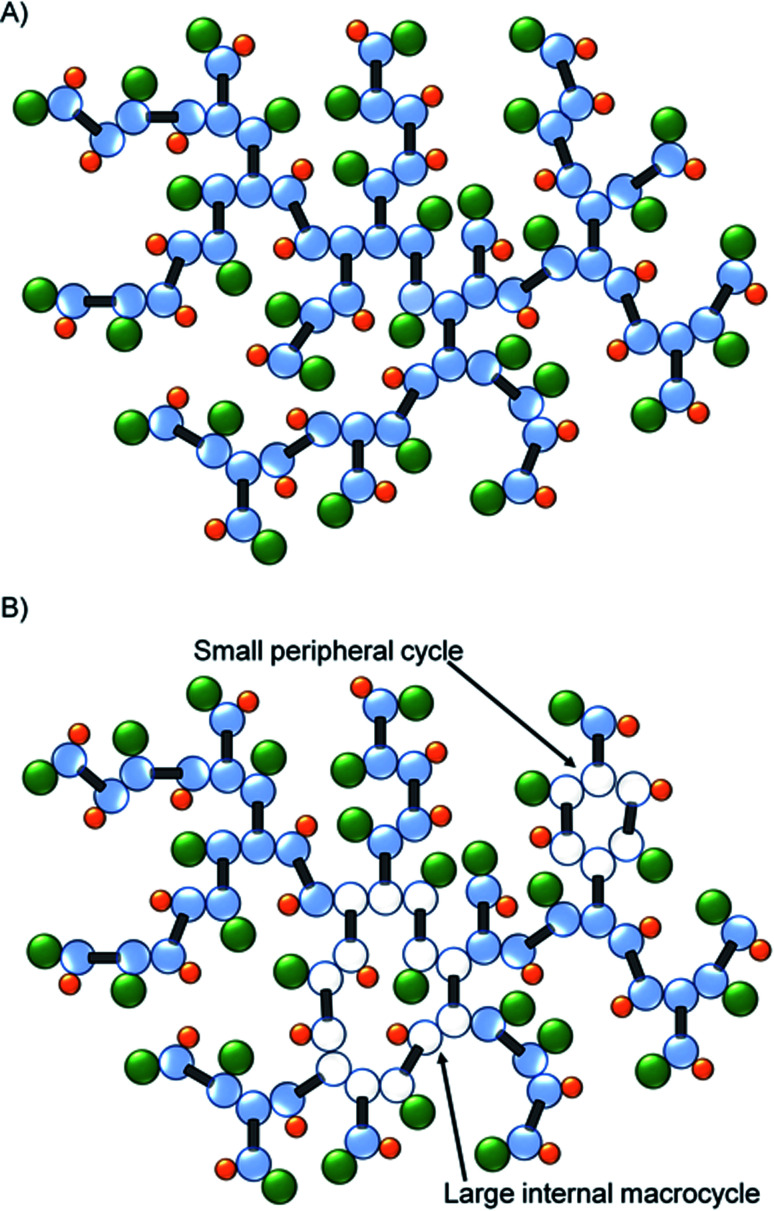
Schematic representation of (A) ideal TBRT polymer architecture, and (B) the formation of macrocyclic substructures within the TBRT polymer.

As mentioned earlier, the identification of macrocyclic structures within branched polymers is often identified by the lack of a key functional or structural group. The decrease in the mole percent of T groups, when coupled to the subsequent increase in resonances for telomer substructures and the decrease in telogen residues (relative to taxogen) at very high molecular weight, suggests significant cyclisation is present in this fraction of the sample, Fig. 5.

Hypothetically, the formation of a macrocycle *via* intramolecular reaction would lead to a reduction in the number of telogens present for any given number of taxogens. For example, as we have already reported, an ideal TBRT structure will contain *n* + 1 telogen residues where *n* = number of taxogens; in the materials presented here, the theoretical number of DDT residues = number of EGDMA residues + 1. The taxogen : telogen ratio would therefore vary as *n* : *n* + 1 and approach a value of 1.00 : 1.00 at high values of *n* (or high molecular weight). As an illustration, Fig. 5A shows an ideal TBRT branched polymer comprising 31 taxogen and 32 telogen residues (taxogen : telogen of 0.97 : 1.00). The formation of a macrocycle *via* intramolecular cyclisation through the reaction of a telogen radical with at least two pendant unreacted vinyl groups during telomerisation, will reduce the theoretical number of telogen residues present in the final branched polymer structure by the number of cycles that are formed (*i.e.* telogens = (*n* + 1) − *c*; where *c* = number of cycles formed). As can be seen in Fig. 5B, macrocycles may be distributed over large regions of the branched polymer structure or impact relatively few taxogen residues; within this schematic example the branched polymer comprises 31 taxogen and 30 telogen residues (taxogen : telogen of 1.03 : 1.00). As stated above, the ideal taxogen : telogen ratio approaches 1.00 : 1.00 at very high molecular weight, therefore values >1.00 are clearly indicative of cyclisation.

Assuming that the observed 1.26 : 1.00 taxogen : telogen ratio determination by ^1^H NMR is not hampered by resonance suppression within these high molecular weight materials, the ratio would suggest as many as 20% of the taxogens may be involved in cycle formation within the highest molecular weight fraction in this case. Importantly, we are not able to observe resonances that directly relate to cycle formation, as with previous reports of branched polymer characterisation from other synthesis strategies,^76^ and we are monitoring the decrease in telogen fragments as a surrogate for the presence of cycles. The formation of cycles during linear telomerisation has, however, been reported previously,^77^ and their presence is not unexpected within branched polymers. Monte Carlo simulations have, for example, shown that extended cyclisation would be a considerable factor within a modified “Strathclyde” polymerisation where the bifunctional monomer is present at very low concentration.^42^ It is possible that the cycles are formed at early stages of the polymerisation and are incorporated into the larger polymer structures through intermolecular reaction; conversely, it is also possible that at relatively high conversion, the low concentration of residual vinyl functionality (either free EGDMA or pendant to larger macromolecules) encourages cycle formation towards the end of the TBRT reaction. Further work will utilise the approaches described here to comprehensively characterise samples from various times points of TBRT reactions and establish the timing of cycle formation, the control of branching and the manipulation of both parameters to optimise TBRT polymer properties.

## Conclusions

TBRT represents a new synthetic strategy for branched polymer synthesis. Importantly, the formation of extended branched macromolecular backbones that would conventionally be formed from step-growth polymerisation approaches offers considerable opportunity, as free radical telomerisation is rapid, cheap and does not require the reaction conditions or reactive chemistries associated with step-growth polymerisation. It is widely recognised that the quantification of branching allows comparison and understanding of property variation across branched polymers made under different conditions or with different starting materials. This report offers such a measurement in the early stages of TBRT development to allow researchers a framework to investigate the scope of TBRT and provide an insight into the structural features within the resulting polymers.

## Conflicts of interest

SRC, PC and SPR are co-inventors on patents that protect the TBRT chemistry; these patents have been licensed to Scott Bader and form the basis of Polymer Mimetics Ltd (Company number 12598928).

## Supplementary Material

RA-011-D1RA03886A-s001

## References

[cit1] Zimm B. H., Stockmayer W. H. (1949). J. Chem. Phys..

[cit2] Mai D. J., Marciel A. B., Sing C. E., Schroeder C. M. (2015). ACS Macro Lett..

[cit3] Tomalia D. A., Baker H., Dewald J., Hall M., Kallos G., Martin S., Roeck J., Ryder J., Smith P. (1985). Polym. J..

[cit4] Newkome G. R., Yao Z., Baker G. R., Gupta V. K. (1985). J. Org. Chem..

[cit5] Hawker C. J., Fréchet J. M. J. (1990). J. Am. Chem. Soc..

[cit6] Carothers W. H. (1936). Trans. Faraday Soc..

[cit7] Mann J. L., Rossi R. L., Smith A. A. A., Appel E. A. (2019). Macromolecules.

[cit8] Everaers R., Grosberg A. Y., Rubinstein M., Rosa A. (2017). Soft Matter.

[cit9] Kim Y. H., Young H., Webster O. W. (1992). Macromolecules.

[cit10] Malmström E., Johansson M., Hult A. (1995). Macromolecules.

[cit11] Froehling P. J. (2004). J. Polym. Sci., Part A: Polym. Chem..

[cit12] Cook A. B., Perrier S. (2020). Adv. Funct. Mater..

[cit13] Jeon I. Y., Noh H. J., Baek J. B. (2018). Molecules.

[cit14] Gao Y., Zhou D., Lyu J., Sigen A., Xu Q., Newland B., Matyjaszewski K., Tai H., Wang W. (2020). Nat. Rev. Chem..

[cit15] Kaiser T., Frey H. (2020). Polymer.

[cit16] Seo S. E., Hawker C. J. (2020). Macromolecules.

[cit17] Zheng Y., Li S., Weng Z., Gao C. (2015). Chem. Soc. Rev..

[cit18] Voit B. I., Lederer A. (2009). Chem. Rev..

[cit19] Gao Y., Zhou D., Lyu J., Sigen A., Xu Q., Newland B., Matyjaszewski K., Tai H., Wang W. (2020). Nat. Rev. Chem..

[cit20] Kaiser T., Frey H. (2020). Polymer.

[cit21] Frey H., Haag R. (2002). J. Biotechnol..

[cit22] Wilms D., Stiriba S.-E., Frey H. (2010). Acc. Chem. Res..

[cit23] von Harpe A., Petersen H., Li Y., Kissel T. (2000). J. Controlled Release.

[cit24] O'Brien N., McKee A., Sherrington D. C., Slark A. T., Titterton A. (2000). Polymer.

[cit25] Weaver J., Williams R., Findlay P., Royles B., Cooper A. I., Rannard S. P. (2008). Soft Matter.

[cit26] Isaure F., Cormack P. A. G., Sherrington D. C. J. (2003). Mater. Chem..

[cit27] Bannister I., Billingham N. C., Armes S. P., Rannard S. P., Findlay P. (2006). Macromolecules.

[cit28] Alhilfi T., Chambon P., Rannard S. P. (2020). J. Polym. Sci..

[cit29] Butun V., Bannister I., Billingham N. C., Sherrington D. C., Armes S. P. (2005). Macromolecules.

[cit30] Li Y. T., Armes S. P. (2005). Macromolecules.

[cit31] Slater R. A., McDonald T. O., Adams D. J., Draper E. R., Weaver J. V. M., Rannard S. P. (2012). Soft Matter.

[cit32] Hatton F. L., Tatham L. M., Tidbury L. R., Chambon P., He T., Owen A., Rannard S. (2015). Chem. Sci..

[cit33] Liu B., Kazlauciunas A., T Guthrie J., Perrier S. (2005). Macromolecules.

[cit34] Fréchet J. M. J., Henmi M., Gitsov I., Aoshima S., Leduc M. R., Grubbs R. B. (1995). Science.

[cit35] Alfurhood J. A., Bachler P. R., Sumerlin B. S. (2016). Polym. Chem..

[cit36] Hutchings L. R. (2008). Soft Matter.

[cit37] Matmour R., Gnanou Y. (2008). J. Am. Chem. Soc..

[cit38] Atkins C. J., Seow D. K., Burns G., Town J. S., Hand R. A., Lester D. W., Cameron N. R., Haddleton D. M., Eissa A. M. (2020). Polym. Chem..

[cit39] McEwan K. A., Haddleton D. M. (2011). Polym. Chem..

[cit40] Koh M. L., Konkolewicz D., Perrier S. (2011). Macromolecules.

[cit41] Wang W., Zheng Y., Roberts E., Duxbury C. J., Ding L., Irvine D. J., Howdle S. M. (2007). Macromolecules.

[cit42] Cassin S. R., Chambon P., Rannard S. P. (2020). Polym. Chem..

[cit43] Harmon J. , *US Pat.* 2 390 099, 1945

[cit44] Ford T. A. , *US Pat.* 2 394 761, 1946

[cit45] Peterson M. D. and Weber A. G., *US Pat.* 2 395 292, 1946

[cit46] Hanford W. E. , *US Pat.* 2 396 786, 1946

[cit47] Howk B. W. , Roland J. R. and Hoehn H. H., *US Pat.* 2 409 683, 1946

[cit48] Muller E. (1952). Angew. Chem..

[cit49] Boutevin B. (2000). J. Polym. Sci., Part A: Polym. Chem..

[cit50] Kudo R., Tsukamoto T., Nakajo S., Fujimori A., Oishi Y., Shibasaki Y. (2021). Eur. Polym. J..

[cit51] Bannister I., Billingham N. C., Armes S. P. (2009). Soft Matter.

[cit52] Cormack P. A. G., Graham S., Sherrington D. C. (2005). Macromolecules.

[cit53] Hawker C. J., Lee R., Frechet J. M. J. (1991). J. Am. Chem. Soc..

[cit54] Kim Y. H. (1994). Macromol. Symp..

[cit55] Hölter D., Burgath A., Frey H. (1997). Acta Polym..

[cit56] Gaborieau M., Castignolle P. (2011). Anal. Bioanal. Chem..

[cit57] Gaborieau M., Gilbert R. G., Gray-Weale A., Hernandez J. M., Castignolles P. (2007). Macromol. Theory Simul..

[cit58] Tobita H., Hamashima N. (2000). J. Polym. Sci., Part B: Polym. Phys..

[cit59] Kashina A. V., Meleshko T. K., Bezrukova M. A., Yakimansky A. V. (2020). Eur. Polym. J..

[cit60] Lezov A., Gubarev A., Kaiser T., Tobaschus W., Tsvetkov N., Nischang I., Schubert U. S., Frey H., Perevyazko I. (2020). Macromolecules.

[cit61] Murima D., Pasch H. (2020). Macromol. Rapid Commun..

[cit62] Smith W. C., Geisler M., Lederer A., Williams S. K. R. (2019). Anal. Chem..

[cit63] Kambouris P., Hawker C. J. (1993). J. Chem. Soc., Perkin Trans. 1.

[cit64] Zhishan B., Schlüter A. D. (2003). Chem. Commun..

[cit65] Rannard S. P., Davis N. J., Herbert I. (2004). Macromolecules.

[cit66] Turner S. R., Walter F., Voit B. I., Mourey T. H. (1994). Macromolecules.

[cit67] Khalyavina A., Schallausky F., Komber H., Al Samman M., Radke W., Lederer A. (2010). Macromolecules.

[cit68] Feast W. J., Keeney A. J., Kenwright A. M., Parker D. (1997). Chem. Commun..

[cit69] Wang Y.-M., Chang P.-Y., Zhao Z.-F., Wang H.-J. (2016). J. Polym. Res..

[cit70] Chu F., Hawker C. J., Pomery P. J., Hill D. J. T. (1997). J. Polym. Sci., Part A: Polym. Chem..

[cit71] Graff R. W., Wang X., Gao H. (2015). Macromolecules.

[cit72] Cuneo T., Wang X., Shi Y., Gao H. (2020). Macromol. Chem. Phys..

[cit73] Chen D.-X., Gao L.-F., Li X.-H., Tu Y.-F. (2017). Chin. J. Polym. Sci..

[cit74] Hatton F. L., Chambon P., Savage A. C., Rannard S. P. (2016). Chem. Commun..

[cit75] Kamide K., Miyazaki Y., Abe T. (1977). Polym. J..

[cit76] Rosselgong J., Armes S. P., Barton W. R. S., Price D. (2010). Macromolecules.

[cit77] Bertrais H., Boutevin B., Maliszewicz M., Vernet J.-L. (1982). Eur. Polym. J..

