# Supplementary material for: Quantification of branching within high molecular weight polymers with polyester backbones formed by transfer-dominated branching radical telomerisation (TBRT)
Source: RSC Adv. 2021 Jul 12;11(39):24374–80. doi: 10.1039/d1ra03886a (PMC9036642; doi:10.1039/d1ra03886a)
Supplement: RA-011-D1RA03886A-s001 [file RA-011-D1RA03886A-s001.pdf]

Electronic Supplementary Material (ESI) for RSC Advances.
This journal is © The Royal Society of Chemistry 2021

Quantification of branching within high molecular weight polymers with polyester backbones formed by Transfer dominated Branching Radical Telomerisation (TBRT

Savannah Cassin, Sean Flynn, Pierre Chambon and Steve P. Rannard*

*Corresponding author: E-mail: srannard@liv.ac.uk

Supplementary Information:

Materials and Methods

Supplementary Figures 1 to 14

Supplementary Table 1

References

Table of Contents

1. **Materials and Methods**

    1.1 Materials

    1.2 Methods

        1.2.1 Nuclear Magnetic (NMR) Spectroscopy

        1.2.2 Triple Detection-Size Exclusion Chromatography (TD-SEC)

2. **Experimental**

2.1 TBRT of EGDMA with varying equivalents of DDT in ethyl acetate

        2.1.1 Solvent fractionation purification

3. **References**

1. Materials and Methods

1.1 Materials

2, 2'-Azobis(2-methylpropionitrile) (AIBN, 98 %), $CDCl_3$ (99.8 atom% D), $Cl_2CDCDCl_2$ (≥ 99.5 %), Ethylene glycol dimethacrylate (EGDMA, 98 %) were purchased from Sigma Aldrich. Ethyl Acetate (EtOAc, analytical grade), Methanol (MeOH, analytical grade 99.9 %). Tetrahydrofuran (THF, HPLC-grade), Acetone (CH_3COCH_3, reagent grade) were purchased from Fisher. 1-Dodecanethiol (DDT, 98 %) was purchased from Alfa Aesar. All materials were used as received unless otherwise stated.

1.2 Methods

1.2.1 Nuclear magnetic resonance (NMR) spectroscopy

^1H, inverse-gated ^{13}C and Attached Proton Test (APT) experiments at 50 mg/mL were recorded on a Bruker AMX-400 MHz spectrometer. Samples were analysed in deuterated chloroform ($CDCl_3$) at room temperature. Chemical shifts (δ) are reported in parts per million (ppm) relative to the known solvent residual peak (δ = 7.26 ppm). Additional inverse-gated ^{13}C experiments were also performed at 50 ˚C.

1.2.2 Triple Detection Size Exclusion Chromatography (TD-SEC)

All TD-SEC analysis of branched polymers were performed using a Malvern Viscotek instrument using GPCmax VE2001 autosampler, two Viscotek T6000M columns (and a guard column), a refractive index (RI) detector VE3580 and a 270 Dual Detector (light scattering and viscometer) with a mobile phase of THF containing 2 v/v % of triethylamine at 35 °C and a flow-rate of 1 mL/min. All samples were dissolved at 10 mg/mL in the eluent and passed through a 200 nm syringe filter prior to injection (100 μL). From TD-SEC results, M_n, M_w, $Đ$, molecular weight distribution and intrinsic viscosity of the polymers were estimated using the Omnisec 5.12 software. Narrow and broad polystyrene standards (Viscotek, M_w = 105 kg/mol, Đ = 1.022 and M_w = 245 kg/mol, Đ = 2.272 respectively) were used to calibrate the instrument.

2. Experimental

2.1 TBRT of EGDMA with varying equivalents of DDT in ethyl acetate.

In a typical synthesis, EGDMA (5 g, 25.2 mmol, 1 equiv.), DDT (6.01 g, 29.7 mmol, 1.18 equiv.), AIBN (0.124 g, 0.757 mmol) were placed into a 25 mL round-bottomed flask. Ethyl acetate was added (50 wt% based on EGDMA and DDT; 11.01 g, 12.2 mL) and the solution stirred and deoxygenated using a nitrogen purge for 15 minutes. The solution was stirred and left to telomerise at 70 °C for 24 hours. The reaction was terminated by exposure to air and cooled. The solution was precipitated into methanol (1:10 ratio) at room temperature affording a white precipitate. After drying the precipitated sample overnight under high vacuum. A sample of the product was taken for NMR spectroscopic analysis in $CDCl_3$ and for TD-SEC analysis in THF.

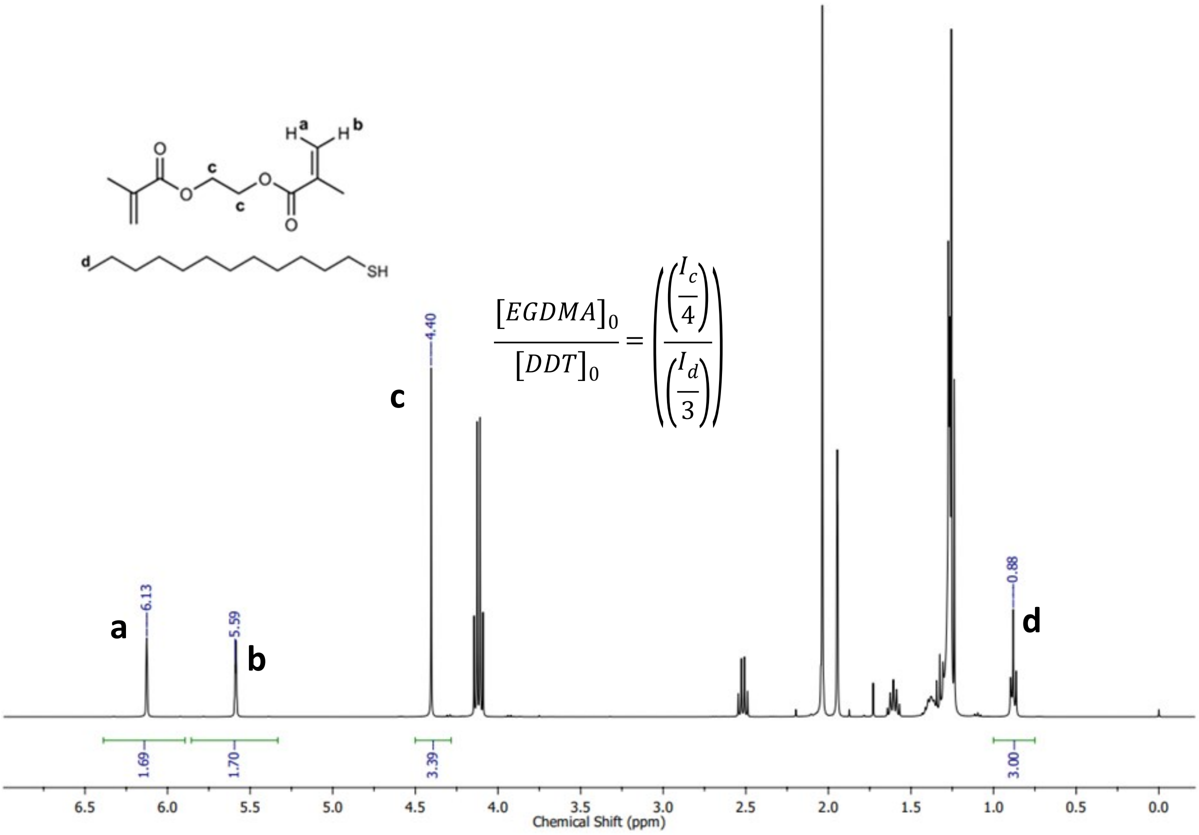

$$\frac{[EGDMA]_0}{[DDT]_0} = \left(\frac{\left(\frac{I_c}{4}\right)}{\left(\frac{I_d}{3}\right)}\right)$$

Figure S1. Quantification of $[EGDMA]_0/[DDT]_0$ by analysis of the reaction, prior to heating, using ^1H NMR spectroscopy (400 MHz, $CDCl_3$). Calculations are based on comparison of the integrals from chemical shifts of EGDMA (c, $-CH_2CH_2-$; 4.0 ppm) and DDT (d. $-CH_3$; 0.88 ppm), following normalisation of the methyl group on DDT at 0.88 ppm to 3H.

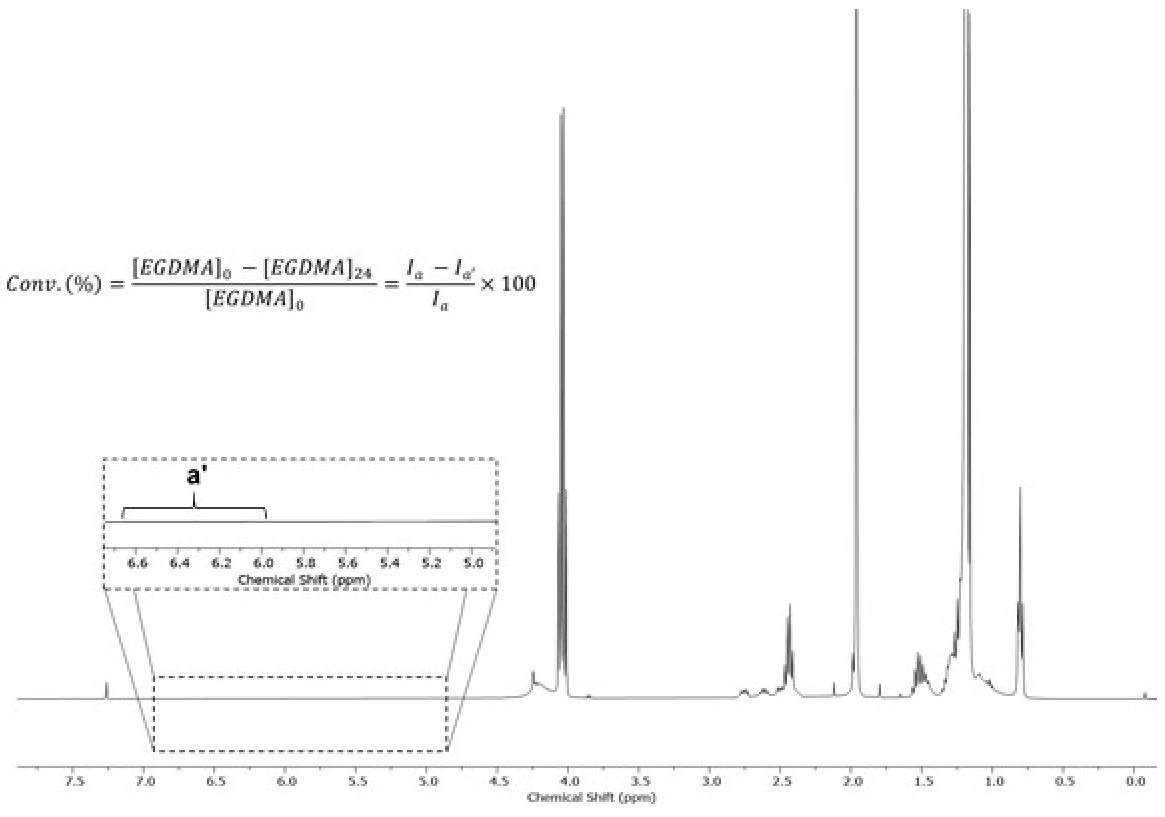

Figure S2. ^{1}H NMR (400 MHz, $CDCl_3$) of $[EGDMA]_0/[DDT]_0 = 0.85$ at t = 24 hours (crude reaction mixture) showing the presence of no vinyl peaks. Calculation of the monomer conversion of EGDMA at 24 hours, using the integrals from the chemical shifts at 5.5 ppm and 6.0 ppm at t = 0 (H_a) and at t = 24 hours ($H_{a'}$), following normalisation of the methyl group on DDT at 0.88 ppm to 3H.

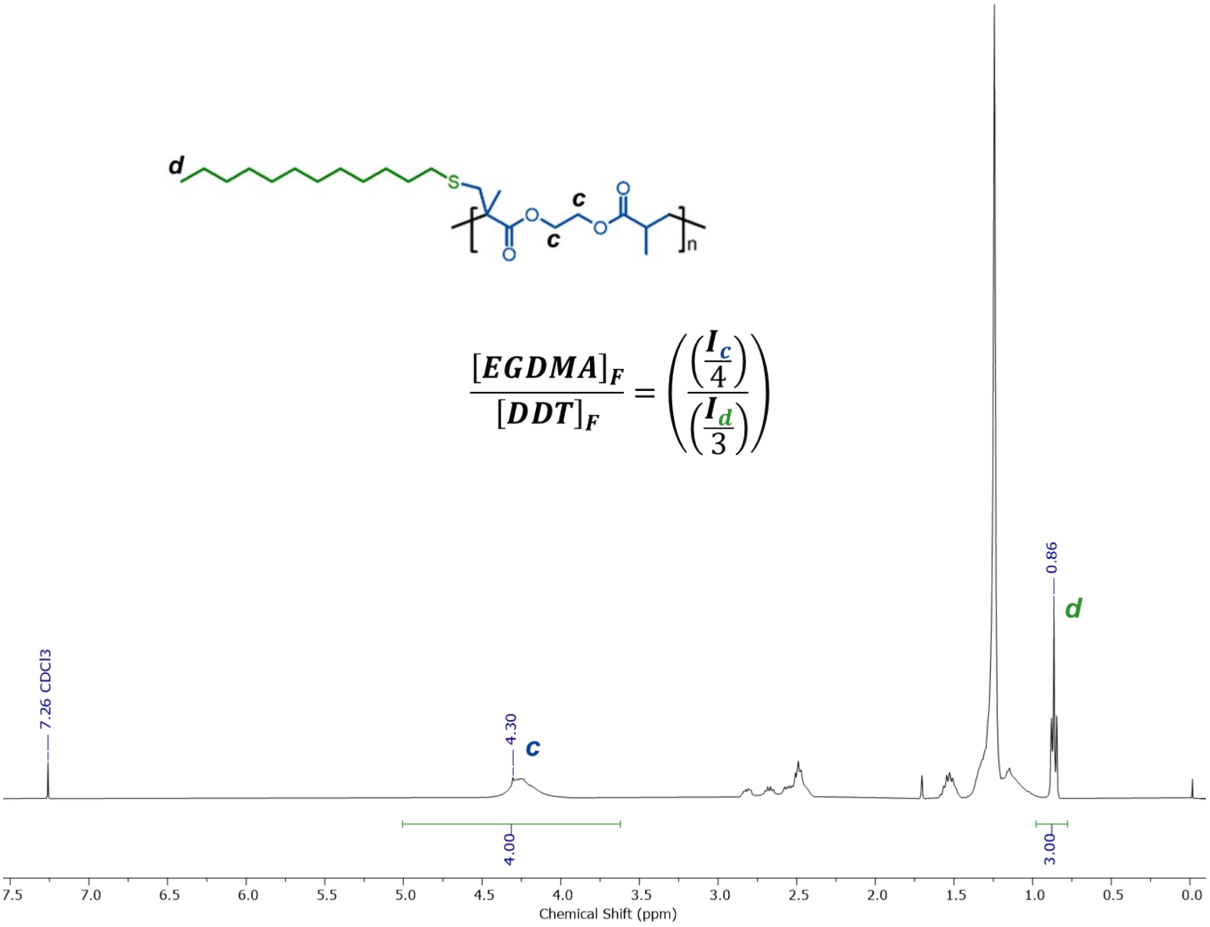

Figure S3. ^{1}H NMR (400 MHz, $CDCl_3$) of the purified sample, $[EGDMA]_F/[DDT]_F = 1.00$. Calculation of the ratio of EGMDA to DDT within the purified product based on comparison of the integrals from chemical shifts of EGDMA (c, $-CH_2CH_2-$, 4.30 ppm) and DDT (d, $-CH_3$, 0.86 ppm), following normalisation of the methyl group on DDT at 0.86 ppm to 3H.

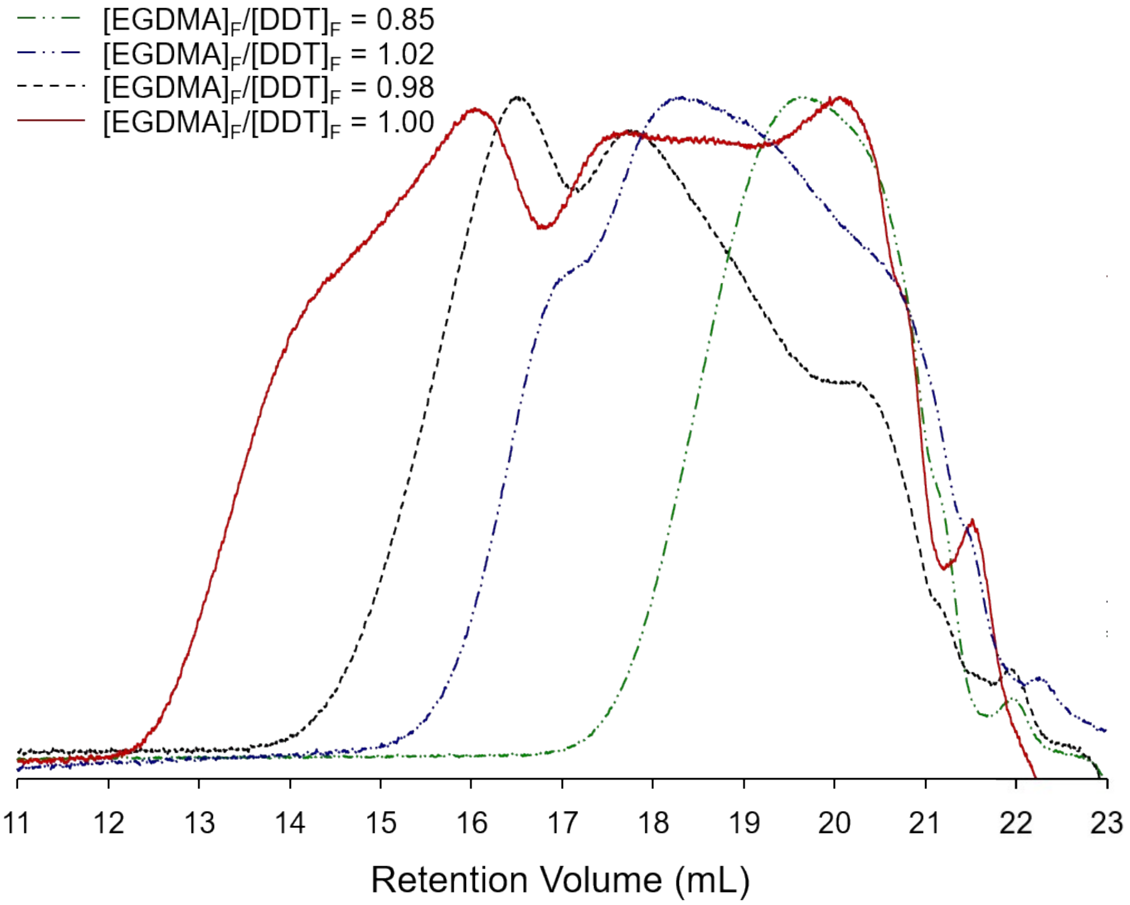

Figure S4. Overlay of RI traces obtained by TD-SEC analysis of the purified samples (manuscript Table 1)

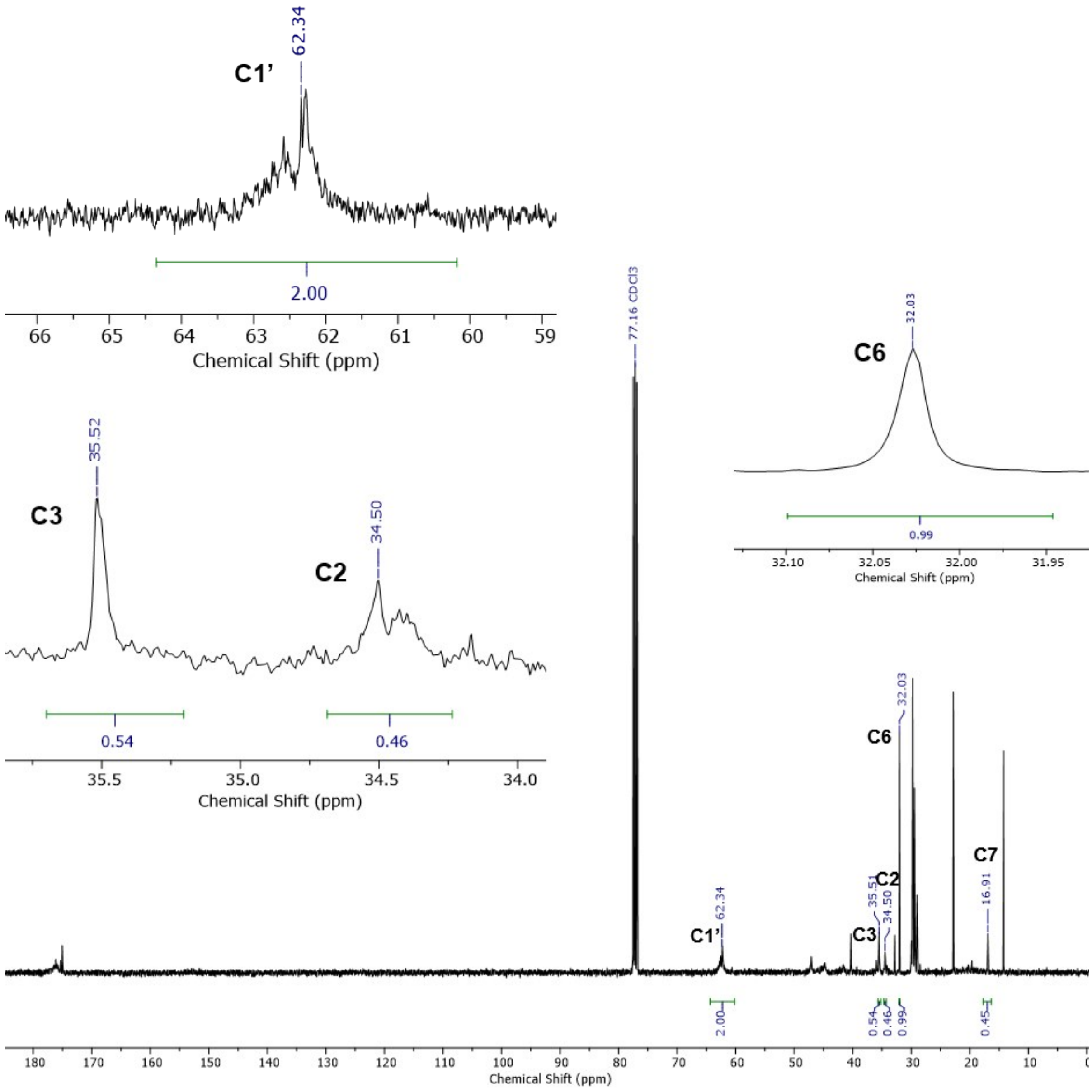

Figure S5. Inverse-gated ^{13}C NMR (100 MHz, $CDCl_3$) of the purified polymer sample with a starting ratio of $[EGDMA]_0/[DDT]_0 = 0.50$. Peaks corresponding to the total EGDMA (C1; 62.34 ppm), terminal carbons (C2; 34.50 ppm and C7; 16.91 ppm), linear carbons (C3; 35.51 ppm) and the total DDT (C6; 32.03 ppm) are labelled.

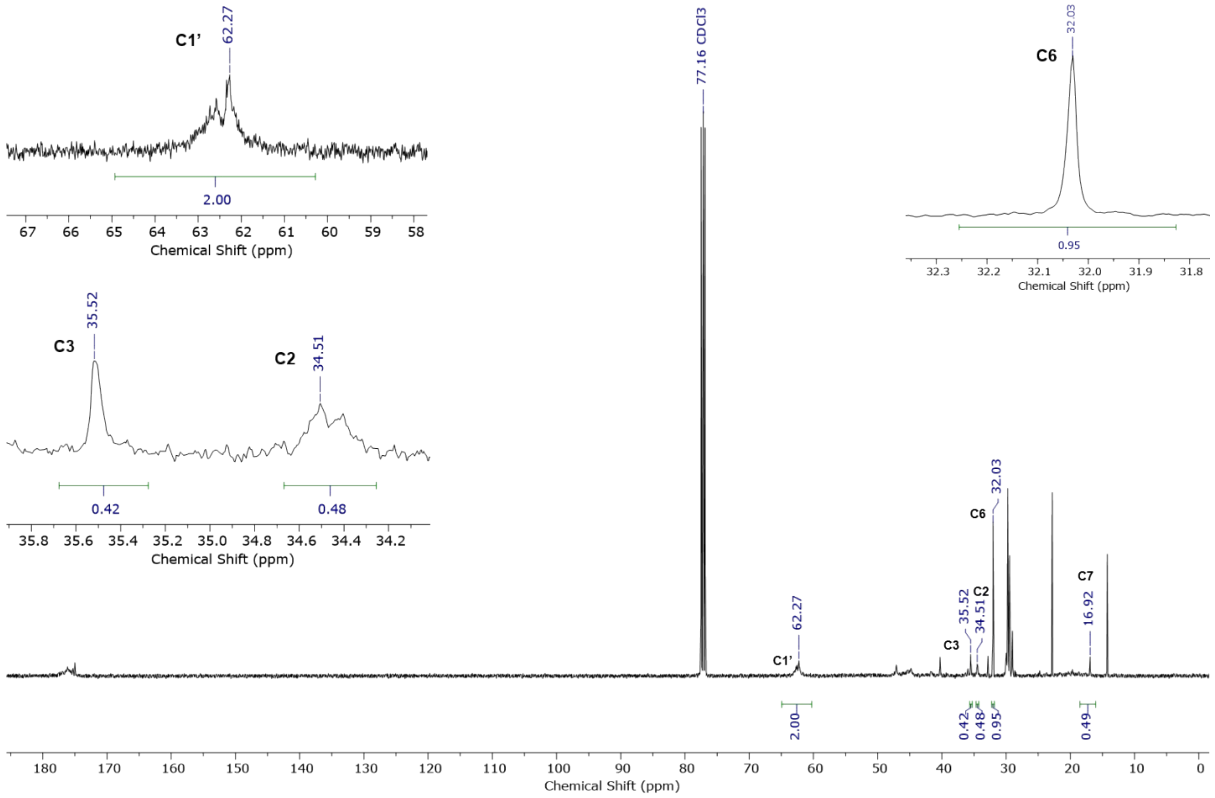

Figure S6. Inverse-gated ^{13}C NMR **(**100 MHz, $CDCl_3$) of the purified polymer sample with a starting ratio of $[EGDMA]_0/[DDT]_0 = 0.75$. Peaks corresponding to the total EGDMA (C1; 62.27 ppm), terminal carbons (C2; 34.51 ppm and C7; 16.92 ppm), linear carbons (C3; 35.52 ppm) and the total DDT (C6; 32.03 ppm) are labelled.

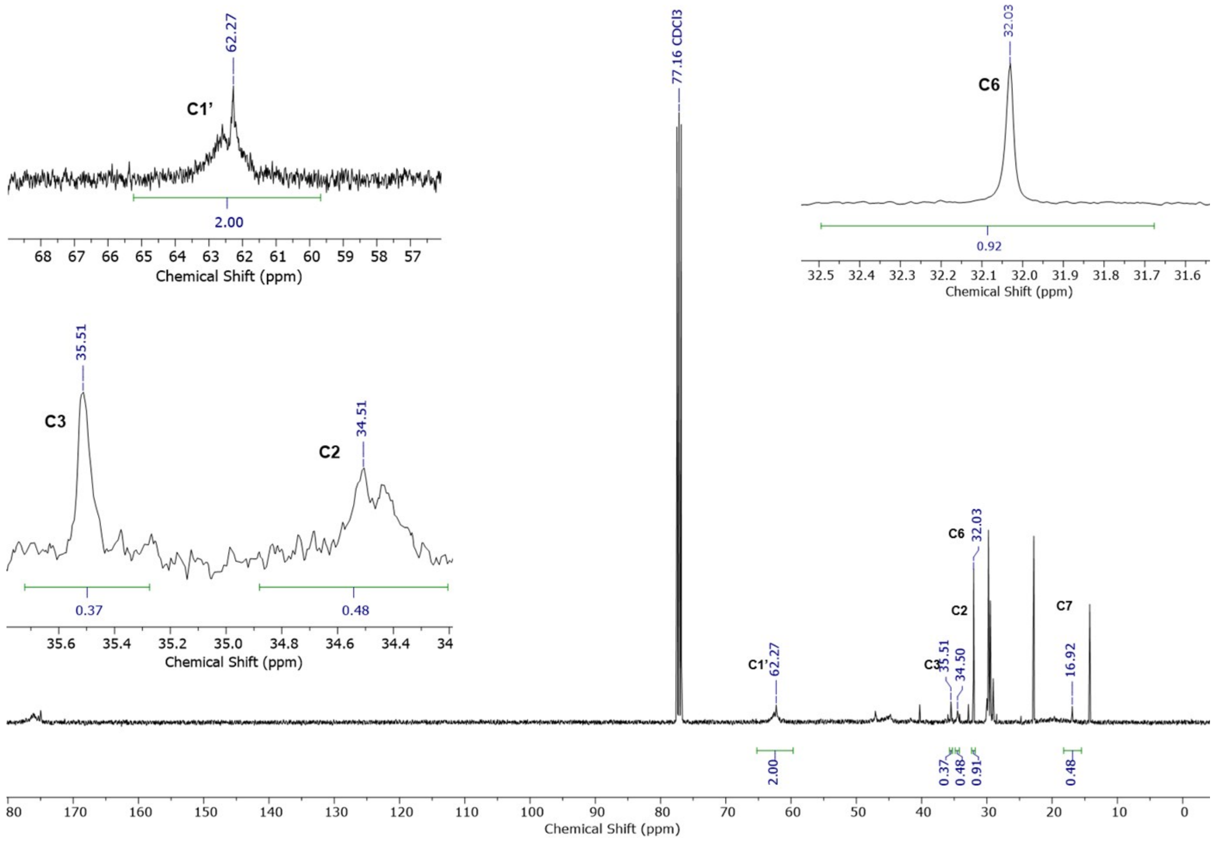

Figure S7. Inverse-gated ^{13}C NMR **(**100 MHz, $CDCl_3$) of the purified polymer sample with a starting ratio of $[EGDMA]_0/[DDT]_0 = 0.80$. Peaks corresponding to the total EGDMA (C1; 62.27 ppm), terminal carbons (C2; 34.50 ppm and C7; 16.92 ppm), linear carbons (C3; 35.51 ppm) and the total DDT (C6; 32.03 ppm) are labelled.

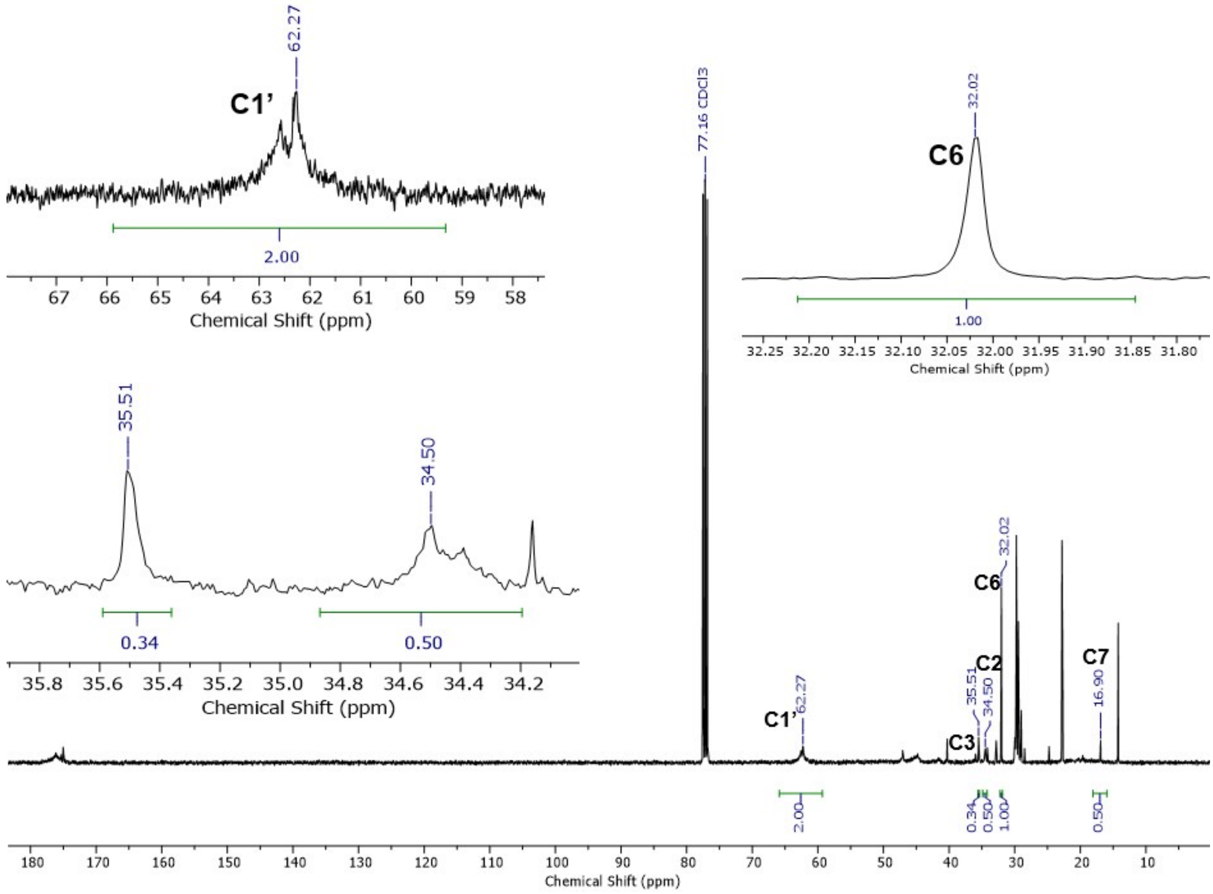

Figure S8. Inverse-gated ^{13}C NMR (100 MHz, $CDCl_3$) of the purified polymer sample with a starting ratio of $[EGDMA]_0/[DDT]_0 = 0.85$. Peaks corresponding to the total EGDMA (C1; 62.27 ppm), terminal carbons (C2; 34.50 ppm and C7; 16.90 ppm), linear carbons (C3; 35.51 ppm) and the total DDT (C6; 32.02 ppm) are labelled.

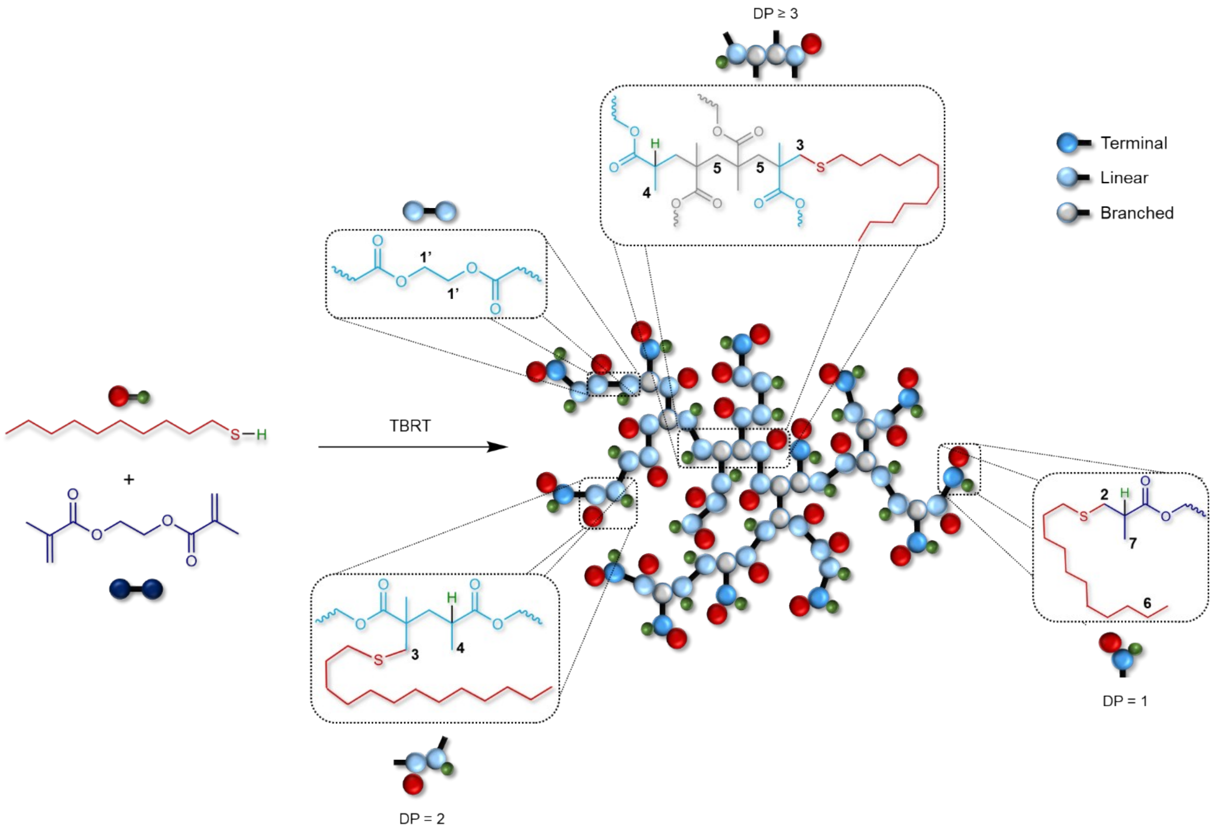

Figure S9. Schematic representation of the structural components within the branched polyester product, highlighting terminal, linear and branched units.

$$\left(I_{C_{1'}} - I_{C_2}\right) - \left(I_{C_3} \cdot 2\right)$$

Equation S1. Calculation of the mol fraction of branched structural sub-units within the purified HBPs. Calculations are based on the subtraction of the integrals from the terminal carbons (C2; 34.50 ppm) from the total EGDMA (C1; 62.34 ppm) and linear carbons (C3; 35.51 ppm).

2.1.1 Solvent fractionation purification

A solvent fractionation was conducted on the purified $[EGDMA]_F/[DDT]_F = 1.00$ sample. The purified sample was dissolved in THF (100 mg mL^{-1}) and acetone (anti-solvent) was added dropwise (40 mL) at ambient temperature. The material that precipitated under these conditions was collected and dried *in vacuo* at 40 °C and characterised via ^1H NMR in $CDCl_3$ (Supplementary Figure 11) and triple detection SEC using THF/TEA eluent (98/2 v/v %) using a narrow and broad poly(styrene) standard calibration.

Table S1. TD-SEC analysis of the TBRT of $[EGDMA]_0/[DDT]_0 = 0.85$ via different purification methods.

	**^1H NMR ($CDCl_3$)**	**TD-SEC (THF)**				
Recovery site	$[EGDMA]_F/[DDT]_F$	M_w (g mol^{-1})	M_n (g mol^{-1})	Đ	α	dn/dc
Precipitation	1.00	2 350 000	21 381	110	0.348	0.087
Solvent Fractionation	1.26	6 557 000	1 102 000	5.95	0.433	0.086

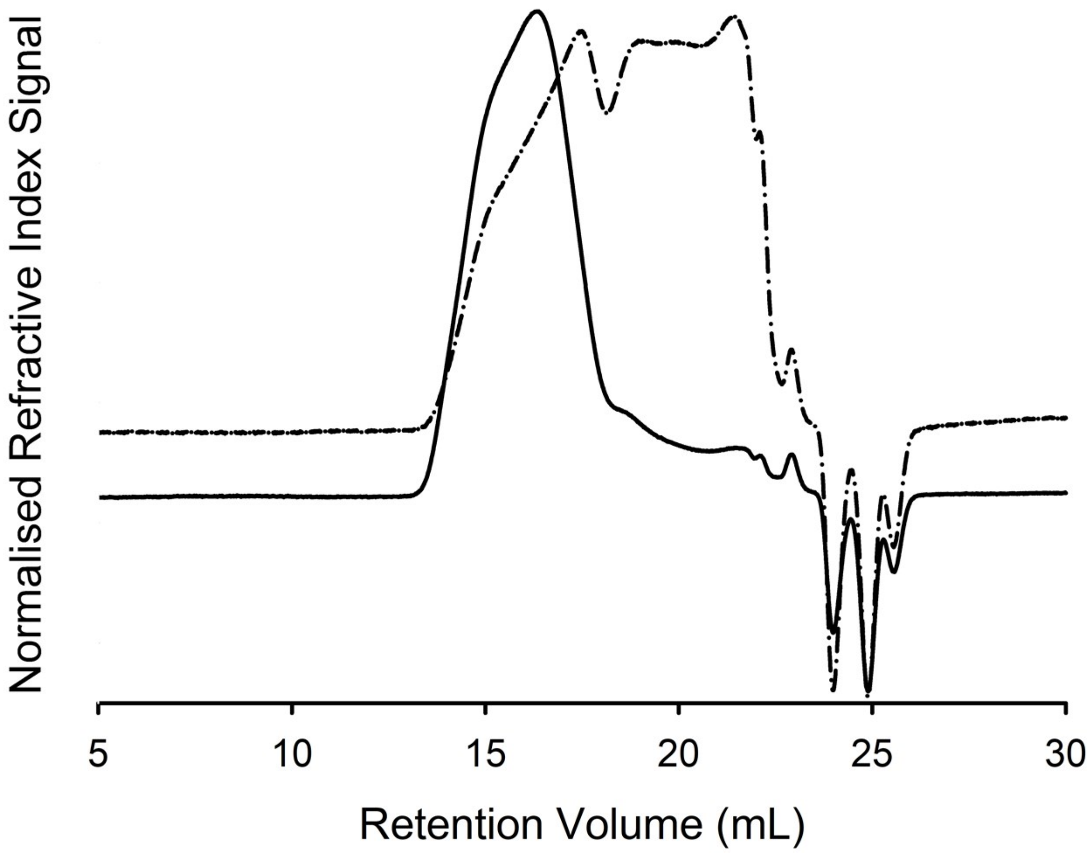

Figure S10. Overlay of RI traces obtained by TD-SEC analysis (THF eluent) of the collected solvent fractionated sample (black solid line) and of the precipitated sample (black dash dost line). Chromatogram also show solvent peaks to confirm correct overlay.

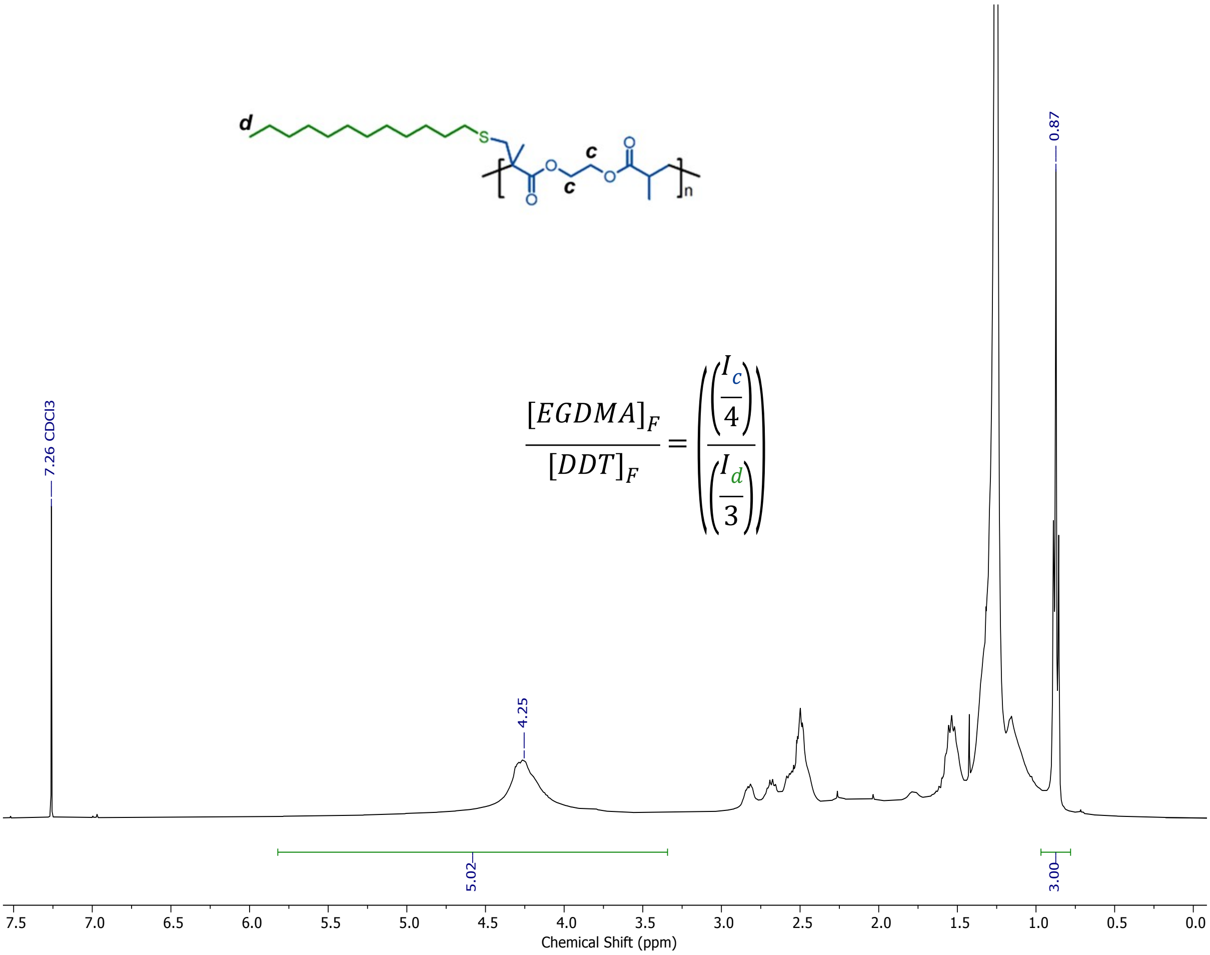

Figure S11. ^{1}H NMR (400 MHz, $CDCl_3$) of the solvent fractionated sample $[EGDMA]_F/[DDT]_F = 1.26$. Calculation of the ratio of EGMDA to DDT within the purified product based on comparison of the integrals from chemical shifts of EGDMA (c, $-CH_2CH_2-$, 4.30 ppm) and DDT (d, $-CH_3$, 0.87 ppm), following normalisation of the methyl group on DDT at 0.87 ppm to 3H.

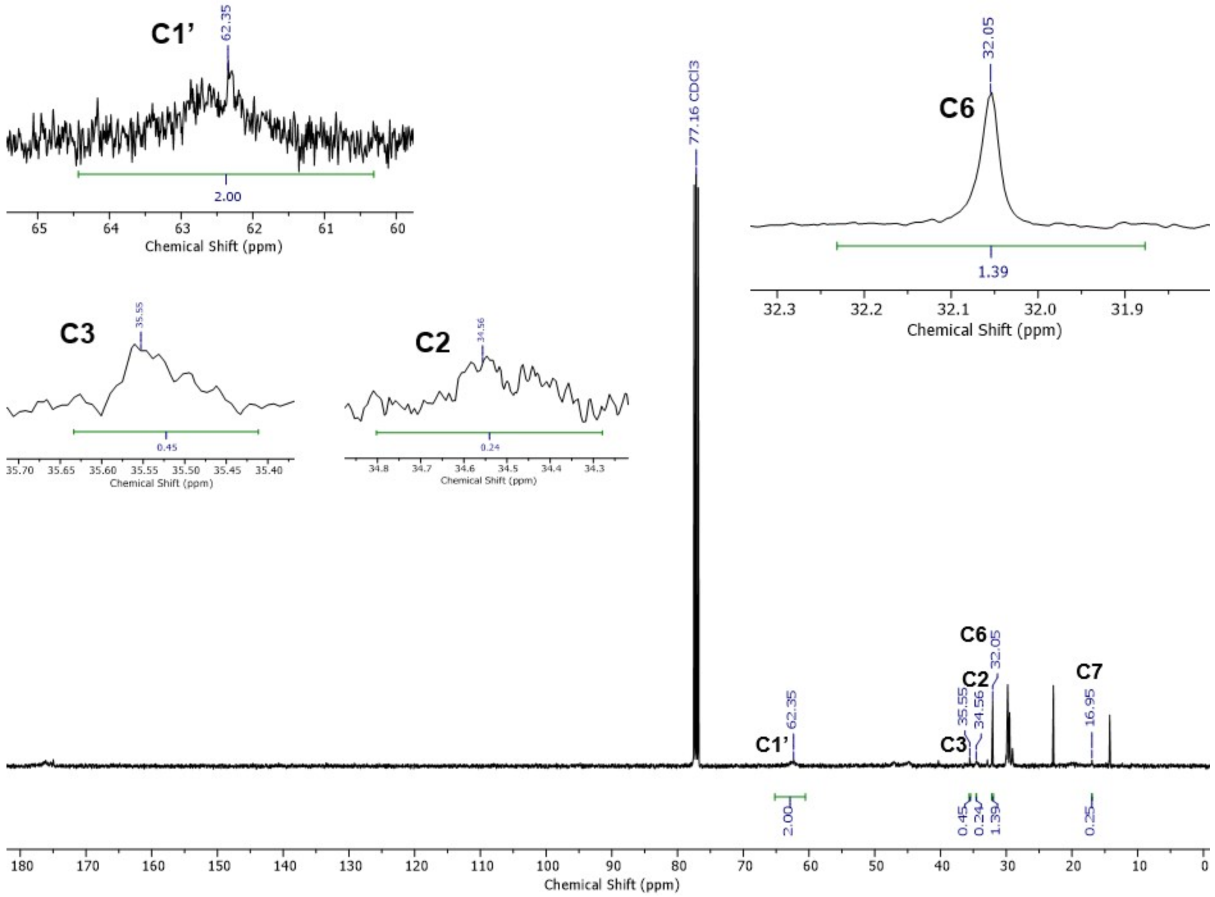

Figure S12. Inverse-gated ^{13}C NMR (100 MHz, $CDCl_3$) of the solvent fractionated sample $[EGDMA]_F/[DDT]_F$ = 1.26. Peaks corresponding to the total EGDMA (C1; 62.35 ppm), terminal carbons (C2; 34.56 ppm and C7; 16.95 ppm), linear carbons (C3; 35.55 ppm) and the total DDT (C6; 32.05 ppm) are labelled.

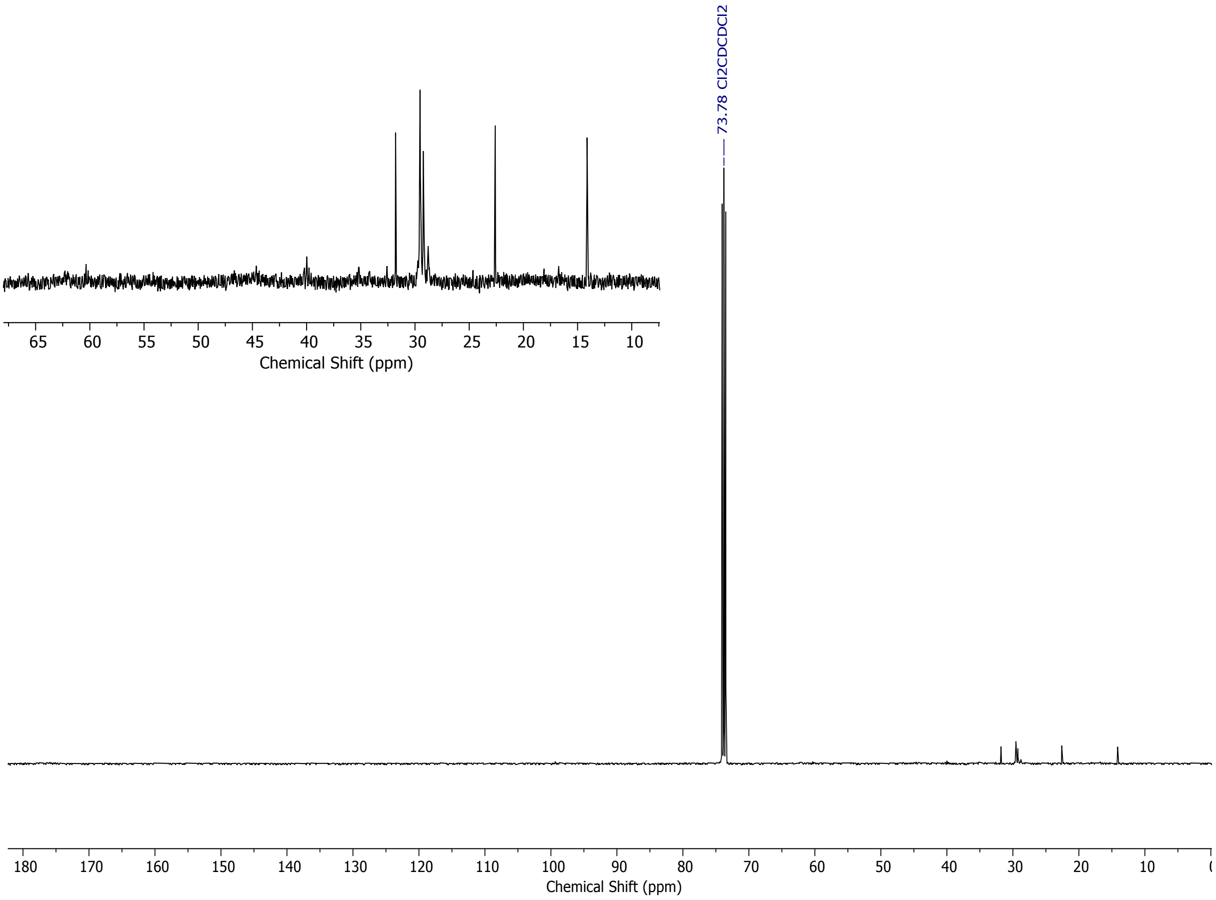

Figure S13. Inverse-gated ^{13}C NMR (100 MHz, $Cl_2CDCDCl_2$) of solvent fractionated $[EGDMA]_F/[DDT]_F$ = 1.26.

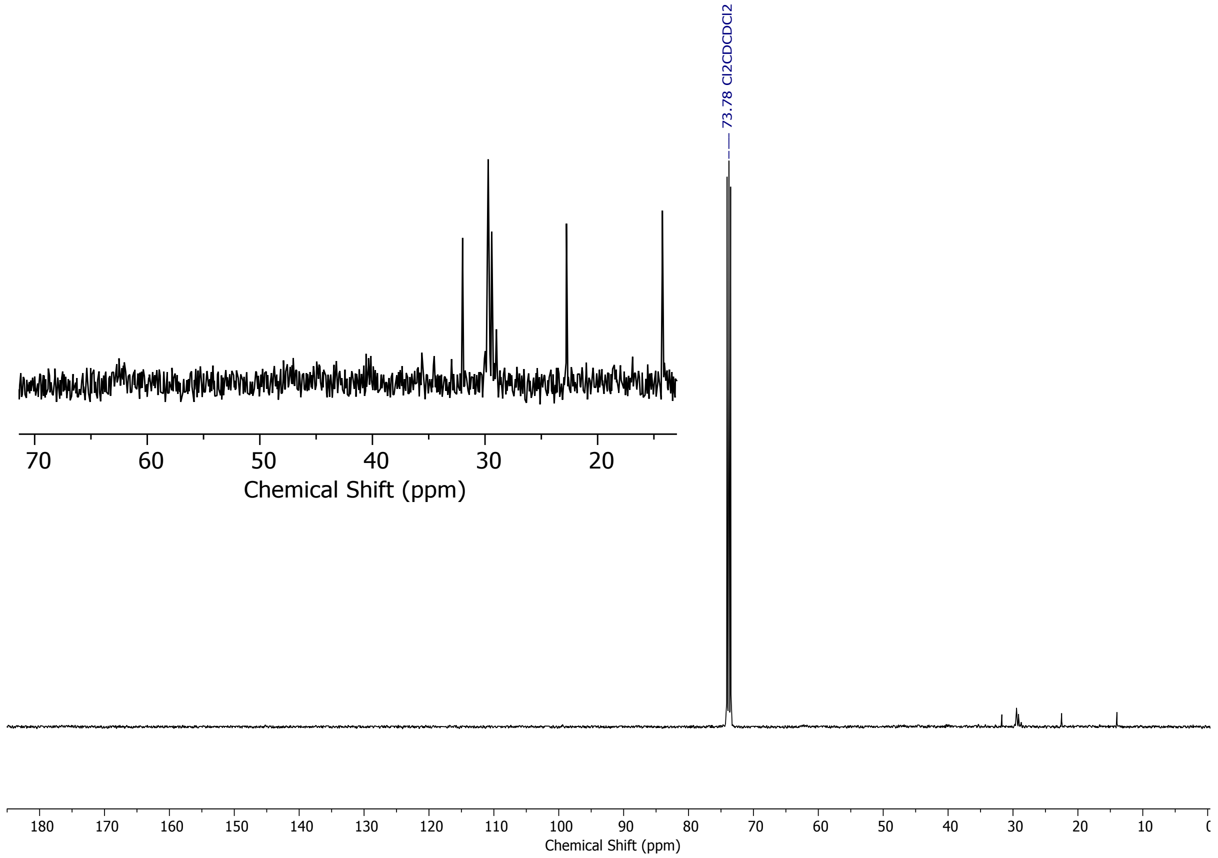

Figure S14. Inverse-gated ^{13}C NMR (100 MHz, $Cl_2CDCDCl_2$) of solvent fractionated $[EGDMA]_F/[DDT]_F$ = 1.26 conducted at 50 °C.
